# Transcriptomic Analyses Shed Light on Critical Genes Associated with Bibenzyl Biosynthesis in *Dendrobium officinale*

**DOI:** 10.3390/plants10040633

**Published:** 2021-03-26

**Authors:** Oluwaniyi Isaiah Adejobi, Ju Guan, Liu Yang, Jiang-Miao Hu, Anmin Yu, Sammy Muraguri, Aizhong Liu

**Affiliations:** 1 Key Laboratory of Economic Plants and Biotechnology, Yunnan Key Laboratory for Wild Plant Resources, Kunming Institute of Botany, Chinese Academy of Sciences, Kunming 650201, China; isaiah@mail.kib.ac.cn (O.I.A.); sammy@mail.kib.ac.cn (S.M.); 2 Skin Care Products Co-Development Center of Dr. Plant, Kunming Institute of Botany, Chinese Academy of Sciences, Kunming 650201, China; yangliu.8355@163.com (L.Y.); hujiangmiao@mail.kib.ac.cn (J.-M.H.); 3 University of the Chinese Academy of Sciences, Beijing 100049, China; 4 Key Laboratory for Forest Resources Conservation and Utilization in the Southwest Mountains of China, Ministry of Education, Southwest Forestry University, Kunming 650224, China; guanju@mail.kib.ac.cn (J.G.); yuanmin@mail.kib.ac.cn (A.Y.); 5 State Key Laboratory of Phytochemistry and Plant Resources in West China, Kunming Institute of Botany, Chinese Academy of Sciences, Kunming 650201, China

**Keywords:** bibenzyl, *Dendrobium officinale*, transcriptome, erianin, gigantol

## Abstract

The *Dendrobium* plants (members of the Orchidaceae family) are used as traditional Chinese medicinal herbs. Bibenzyl, one of the active compounds in *Dendrobium officinale*, occurs in low amounts among different tissues. However, market demands require a higher content of thes compounds to meet the threshold for drug production. There is, therefore, an immediate need to dissect the physiological and molecular mechanisms underlying how bibenzyl compounds are biosynthesized in *D. officinale* tissues. In this study, the accumulation of erianin and gigantol in tissues were studied as representative compounds of bibenzyl. Exogenous application of Methyl-Jasmonate (MeJA) promotes the biosynthesis of bibenzyl compounds; therefore, transcriptomic analyses were conducted between *D. officinale-*treated root tissues and a control. Our results show that the root tissues contained the highest content of bibenzyl (erianin and gigantol). We identified 1342 differentially expressed genes (DEGs) with 912 up-regulated and 430 down-regulated genes in our transcriptome dataset. Most of the identified DEGs are functionally involved in the JA signaling pathway and the biosynthesis of secondary metabolites. We also identified two candidate cytochrome P450 genes and nine other enzymatic genes functionally involved in bibenzyl biosynthesis. Our study provides insights on the identification of critical genes associated with bibenzyl biosynthesis and accumulation in *Dendrobium* plants, paving the way for future research on dissecting the physiological and molecular mechanisms of bibenzyl synthesis in plants as well as guide genetic engineering for the improvement of *Dendrobium* varieties through increasing bibenzyl content for drug production and industrialization.

## 1. Introduction

Plants produce vital secondary metabolites for growth and development and also in response to environmental stresses. These secondary metabolites (such as alkaloids, phenolics, flavonoids, and terpenoids) often accumulate within a specific group of plants and tissues, which play crucial roles in helping plants in defense against various biotic and abiotic stresses [1,2,3]. In particular, these secondary metabolites provide essential resources for new drug innovations, insecticides, and flavors [4,5,6].

The *Dendrobium* plants belong to the family Orchidaceae and are used as traditional Chinese medicinal herbs (referred to as shihu in Mandarin). They are widely distributed across Asia and the Pacific Islands [7]. Previous studies documented the health benefits (antipyretic, ophthalmic, and regulative of the immune system) of *Dendrobium* plants and their contribution to Chinese medicines [8]. Owing to its wealth of active compounds with antitumor and antioxidants functions, *D. officinale* has received tremendous interest in Asian countries. The active medicinal ingredients in *D. officinale* include polysaccharides, alkaloids, phenols, terpenes, flavonoids, and bibenzyl [9,10]. In *Dendrobium* plants, sesquiterpene alkaloid content is the primary measure of its quality and medicinal efficacy [11,12]. Notably, previous studies have found that bibenzyl compounds (belonging to sesquiterpene alkaloids) might be the only bioactive ingredients in *D. officinale* [12,13]. Increasing evidence has shown that bibenzyl compounds are active antitumor agents because of their antioxidant and cell-protective properties [14,15,16,17,18,19]. Bibenzyl compounds have been widely applied to produce several skincare products and medicinal drugs [19,20]. From *Dendrobium* species, previous studies have identified over 190 compounds, including bibenzyl, with erianin and gigantol compounds as the primary representative bio-active compounds in the genus *Dendrobium* [10,17]. Although the biosynthesis of bibenzyl compounds might be complex and conserved in plants, it generally requires the incorporation of dihydro-m-coumaroyl-CoA (1 mol.) and malonyl-CoA (3 mol.) along with the catalyzation of bibenzyl synthase. Four key enzymes are involved in this bibenzyl’s biosynthesis: the initial biosynthesis of dihydro-m-coumaroyl-CoA may start with a molecule of phenylalanine to produce the cinnamate molecule with the catalyzation of ammonia-lyase (PAL). The cinnamate is further incorporated into m-coumaric-CoA with the catalyzation of cinnamate 4-hydroxylase (C4H). Next, dihydro-p-coumaroyl-CoA is synthesized from p-coumaric-CoA with the catalyzation of 4-coumarate: CoA ligase (4CL), and at the same time, dihydro-m-coumaric acid is synthesized from m-coumaric acid with the incorporation of cytochrome P450 (CYP450) [21,22,23,24,25].

CYP450 genes are vital in the regulation of secondary metabolites biosynthesis in plants [26,27]. They participate in the production of defense secondary metabolites [28] such as bibenzyl; therefore, CYP450 expression has a significant effect on bibenzyl quality in plants. The biosynthesis pathway of secondary metabolites involves various physiological factors and regulatory modifications in different plants that have developed their strategies in response to environmental changes or stresses. Usually, the accumulation of secondary metabolites (such as bibenzyl compounds) is low among tissues [5]. However, market demands require a higher content of bibenzyl compounds in *D. officinale* tissues to meet the threshold for drug-making. There is, therefore, an immediate need to dissect the physiological and molecular mechanisms underlying how bibenzyl compounds are biosynthesized in *D. officinale* tissues. The identification of rate-limiting enzymes or regulatory factors, which are responsible for the biosynthesis of bibenzyl compounds, is thus also an area in need of further exploration. It is exceedingly helpful to use genetic engineering and molecular modification techniques to create improved varieties to meet commercial needs. Investigating the physiological and molecular basis of the accumulation of bibenzyl compounds is an essential prerequisite to understanding molecular and genetic factors that regulate the biosynthesis of bibenzyl compounds in *D. officinale* tissues. There is an urgent need for a sustainable source of bibenzyl derived from plants, and *D. officinale* has excellent potential for the production of these bibenzyl compounds. Still, the physiological and molecular mechanisms underlying the biosynthesis of bibenzyl biosynthesis in *D. officinale* remain unexplored. Studies have found that jasmonate (JA), a plant-specific signaling molecule, is widely involved in the biosynthesis of diverse secondary metabolites. The exogenous application of MeJA often results in the strong activation of secondary metabolites biosynthesis and has frequently been applied to induce the biosynthesis of secondary metabolites in plants [29].

With the rapid advancement in RNA-seq technology, transcriptomic data offer both a great opportunity and a powerful tool for the discovery of crucial rate-limiting enzyme or regulator genes, which control the production of some secondary metabolites in plants under different conditions [5,30,31]. Based on transcriptomic analysis, several putative rate-limiting genes responsible for the biosynthesis of terpenoids in *Eugenia uniflora* [32], lignin in *Apium graveolens* [33], and flavonoid in *Phyllanthus emblica, Dracaena cambodiana*, and *Solanum viarum* [34,35,36] have been identified. From *Dendrobium* plants, several studies have reported on flavonoid biosynthetic pathway analysis and the gene mining of key enzymes [37], as well as alkaloid biosynthetic pathway analysis and the identification of key genes [12,38,39]. However, the bibenzyl biosynthetic pathway and the potential genes responsible for regulating bibenzyl biosynthesis in *Dendrobium* plants remain underexplored.

In this study, we investigated the accumulation of bibenzyl in various tissues of *D. officinale*. We conducted and characterized the transcriptome of *D. officinale* root to unravel the putative genes involved in the biosynthesis of bibenzyl in *D. officinale*. This study focused on identifying putative genes associated with the bibenzyl biosynthesis in *Dendrobium* plants, which will aid our understanding of unique genes involved in the synthesis of bibenzyl in *D. officinale*, and provide new insights for future research into the molecular mechanisms of the genes involved in bibenzyl biosynthesis.

## 2. **Results**

### 2.1. Investigation of Bibenzyl Accumulation among Tissues

The root tissues had the highest content of erianin and gigantol, which were 2.63 ± 0.69 and 37.01 ± 2.16 µg/g, respectively, followed by the basal stem (0.61 ± 0.01 and 22.67 ± 0.15 µg/g; see Table 1). Within the upper stem, erianin was not detectable, while the content of gigantol was lower compared to the root and the basal stem tissues. However, neither erianin nor gigantol could be detected in the leaf tissues. These results imply that bibenzyl biosynthesis and accumulation mainly occur in the root tissues.

**Figure 1:**
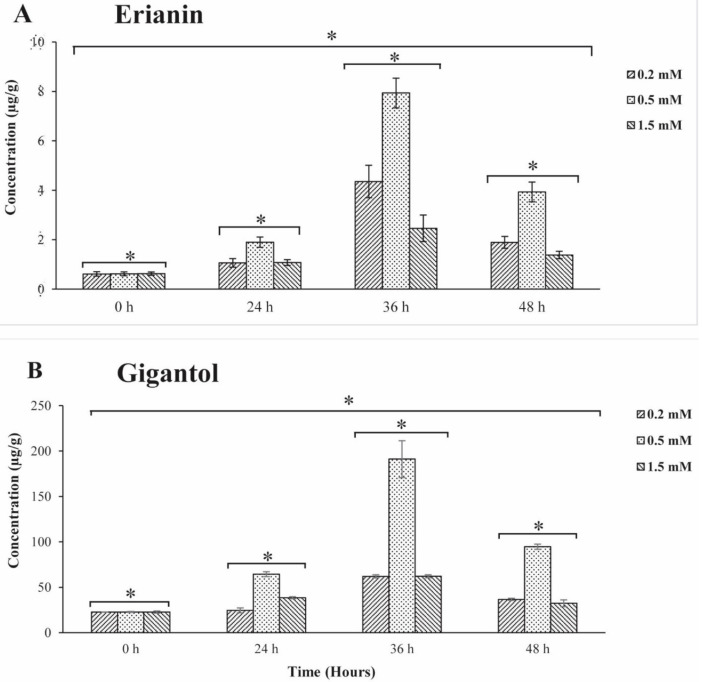
(**A**) Changes of Erianin contents under different exogenous MeJA concentrations treatment using 0.2 mM, 0.5 mM, and 1.5 mM from 0 h to 48 h. (**B**) Changes of Gigantol contents under different exogenous MeJA concentrations treatment using 0.2 mM, 0.5 mM, and 1.5 mM from 0 h to 48 h. Each bar shows the mean ± SE of triplicate assays. The asterisk (*) at the top of the bar indicate significance differences (*p* < 0.05) according to the ANOVA and Tukey tests.

To confirm if exogenous MeJA can induce bibenzyl accumulation, *D. officinale* was treated with MeJA solution at different concentrations. It was observed that the contents of bibenzyl (erianin and gigantol) in root tissues were significantly higher (13.01-fold and 8.43-fold increase, respectively) at 36 hours (Figure 1A,B). However, the 0.5 mM concentration seemed to be optimal to induce the accumulation of bibenzyl in the root tissue. One-way analysis of variance (ANOVA) showed that there were significant differences in the concentration levels of erianin and gigantol with time (*p*-value < 0.05). There was also a significant difference of erianin and gigantol at each particular time for the different concentration. These results clearly show that bibenzyl accumulation was significantly induced at 36 hours by exogenous MeJA in the root tissues.

### 2.2. Transcriptome Sequencing Datasets

Based on the above results, *D. officinale* was treated with MeJA (0.5 mM) while the global transcriptomic changes were reviewed after 24-h treatment compared to the controls (CT). With this, we constructed two cDNA libraries (control group [CT] and treatment group [MJ] for transcriptome sequencing. The raw sequencing data were deposited in the NCBI Sequence Read Archive (SRA) database under the accession numbers SRR9866323 and SRR9866324. In total, 83.21 and 82.62 million raw reads were generated from the CT and MJ libraries, and the Q30 percentages (sequencing error rate < 0.1%) were 95.06% and 95.40%, respectively (Supplementary Table S1). In total, 81.28 and 81.05 million high-quality clean reads were obtained from the two libraries, while 61.50 and 47.57 million reads were uniquely mapped to the reference genome, respectively (Supplementary Table S1). Based on the two transcriptomic datasets, a total of 23,131 genes were annotated.

### 2.3. Identification of Differentially Expressed Genes

**Figure 2:**
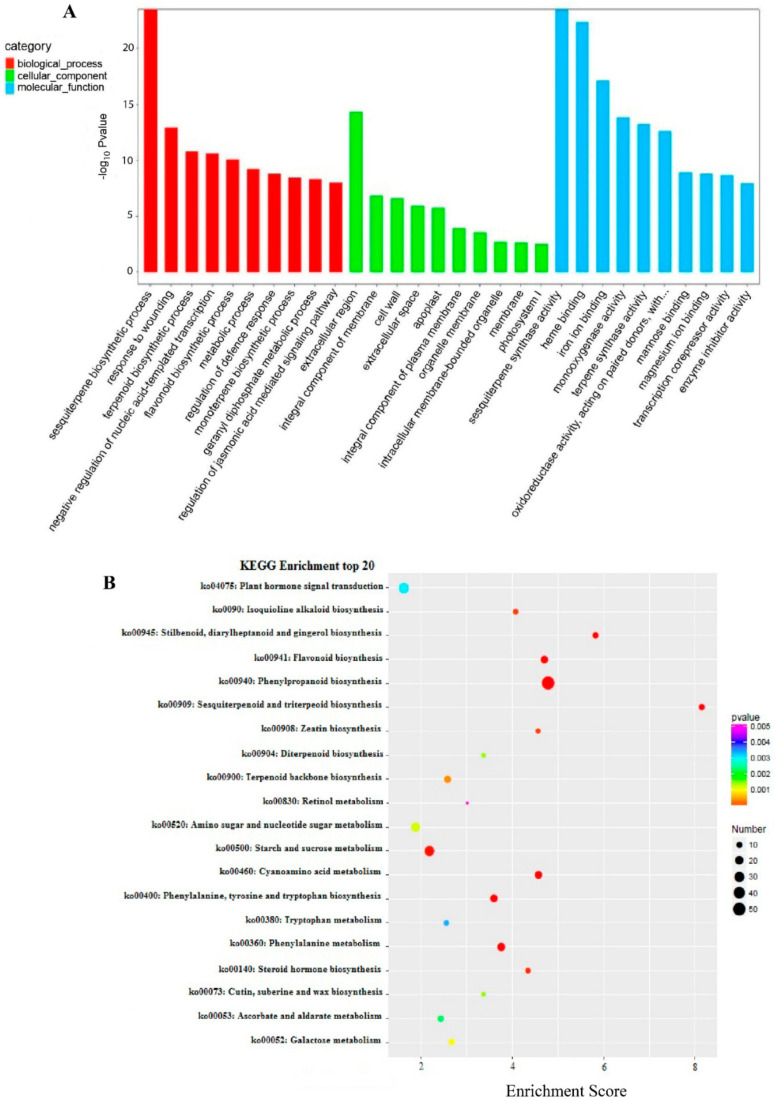
(**A**): Functional GO enrichment analysis for the identified DEGs. GO enrichment analysis of DEGs identified from RNA-Seq analysis. The red, green, and blue bars represent the biological process, cellular component, and molecular function. (**B**): The top 20 pathways within KEGG analysis. KEGG pathway enrichment analysis of DEGs identified from RNA-Seq analysis. The different colors represent the p-value, ranging from 0.001 to 0.005, while the black circle represents gene number. GO: Gene Ontology; DEG: differentially expressed genes; KEGG: Kyoto Encyclopedia of Genes and Genomes.

**Figure 3:**
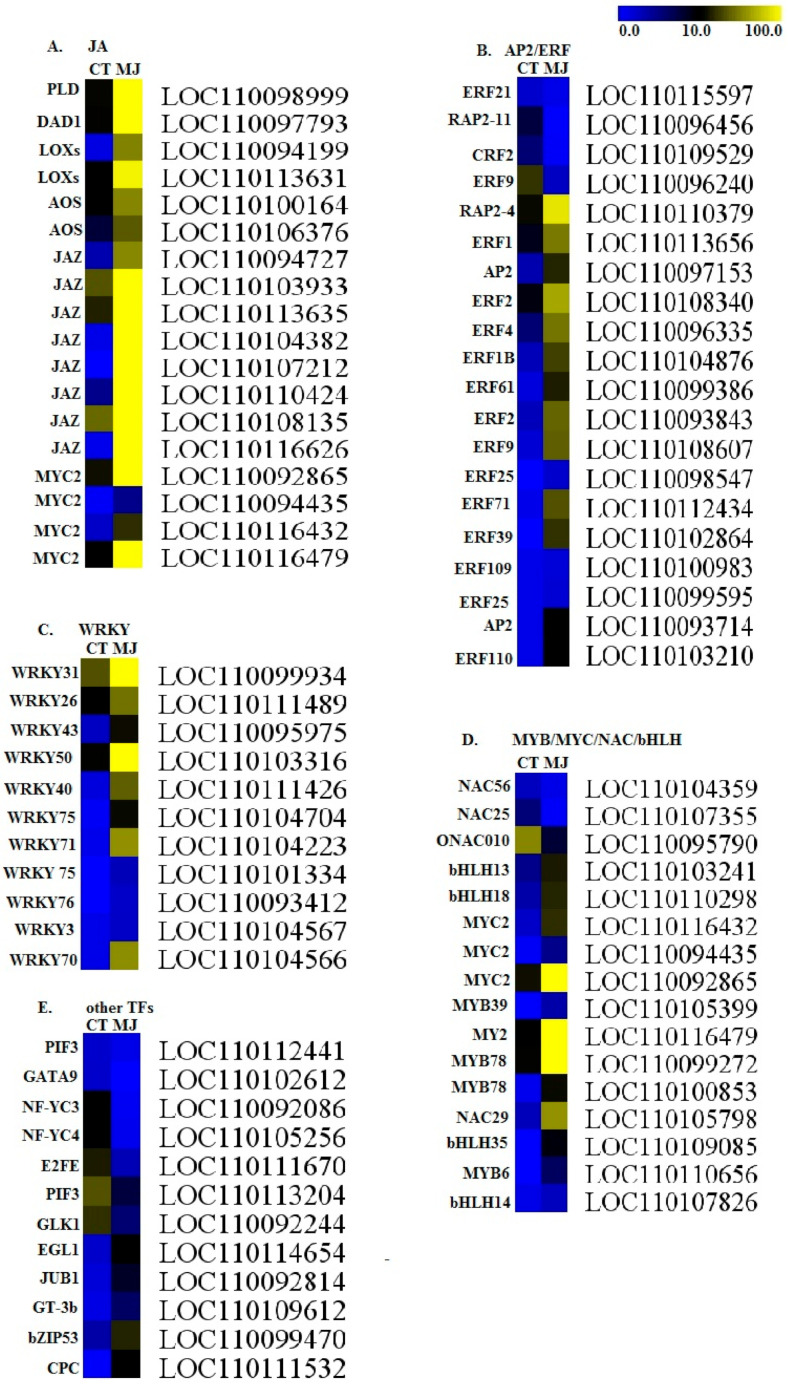
Identification of differentially expressed genes involved in (**A**) the JA signal pathway and (**B**–**E**) TFs involved in bibenzyl compounds biosynthesis. CT signifies the control group, while MJ signifies the treated group. Yellow or blue represents up-regulation or down-regulation, respectively, and black represents the genes at background levels. Scale bar represents fold changes of DEGs expression.

To identify putative genes associated with bibenzyl biosynthesis in *D. officinale*, we analyzed the differentially expressed genes (DEGs) between the two sequenced datasets. Parameters of the False Discovery Rate (FDR) were set at <0.05 and |log2 Fold change| >2 for identifying DEGs. In total, 1324 DEGs, consisting of 912 up-regulated and 430 down-regulated DEGs, were identified compared to the Control (Supplementary Figure S1). To understand these DEGs’ possible functions, we performed Gene Ontology (GO) enrichment analysis for the identified DEGs. We found that these induced DEGs were significantly enriched in GO terms related to the diverse processes of secondary metabolites, including sesquiterpene biosynthesis, response to wounding, flavonoid biosynthesis, regulation of defense response, and regulation of jasmonic acid-mediated signaling pathway (Figure 2A). Furthermore, all DEGs were mapped to terms in the Kyoto Encyclopedia of Genes and Genomes (KEGG) database to search for enriched genes involved in secondary metabolic or signal transduction pathways. In total, 20 pathways with *p*-value < 0.05 were significantly enriched under MeJA treatment (Figure 2B). Notably, some specific enriched DEGs were observed in the pathways of plant hormone signal transduction, phenylpropanoid biosynthesis, flavonoid biosynthesis, and so on. Particularly, all genes involved in the JA signal pathway were up-regulated (Figure 3A), including the LOC110098999 encoding phospholipase D (PLD), the LOC110097793 encoding phospholipase A1 (DAD1), the LOC110113631/LOC110094199 encoding lipoxygenase (LOX), and the LOC110100164/LOC110106376 encoding allene oxide synthase (AOS). Also, eight and four DEGs identified as JAZ and MYC2 in the JA signal pathway were significantly up-regulated under MeJA treatment.

A total of 59 transcription factors (TFs) of DEGs were identified among the control and MeJA data. These include 45 up-regulated and 14 down-regulated TFs (Figure 3B–E). The most abundant TF family was the APELATA2/ethylene response factor (AP2/ERF) superfamily (20 TFs), followed by WRKY (11 TFs), MYB (4 TFs), MYC (4 TFs), NAC (4 TFs), BHLH (4 TFs), and another 12 TFs. Among the 20 AP2/ERF DGEs, 16 AP2/ERF TFs were up-regulated, and only 4 AP2/ERF TFs were down-regulated. All identified WRKY TFs were up-regulated (Figure 3C) under MeJA treatment.

### 2.4. Identification of Candidate Genes Involved in Bibenzyl Biosynthesis

**Figure 4:**
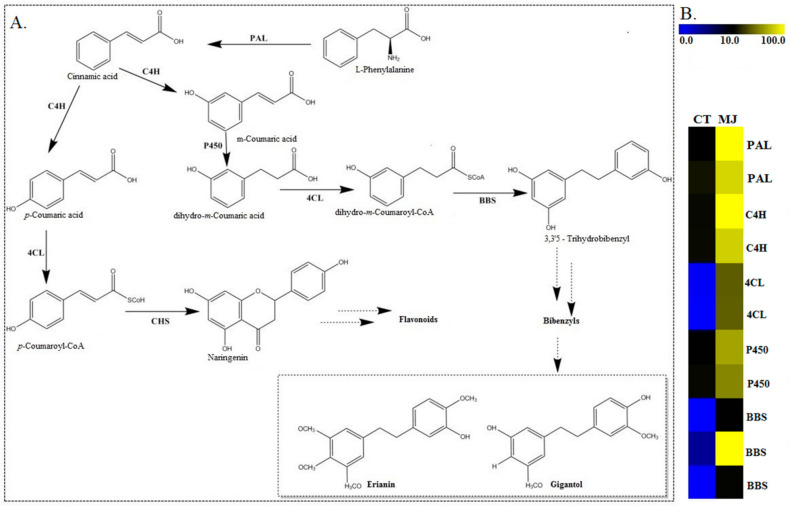
**(A**): Putative bibenzyl biosynthesis pathways and identification of differentially expressed genes involved in bibenzyl biosynthesis. PAL: phenylalanine ammonia-lyase, C4H: trans-cinnamate 4-monooxygenase, P450: cytochrome P450s, 4CL: 4-coumarate-CoA ligase 1,2-like, and BBS: bibenzyl synthase (or bibenzyl synthase-like). (**B**): Changes in the expression of the identified DEGs involved in bibenzyl biosynthesis. CT signifies the control group, while MJ signifies the treated group. Yellow or blue represents up-regulation or down-regulation, respectively, and black represents the genes at background levels. Scale bar represents fold changes of DEGs expression.

Bibenzyl, an alkaloid belonging to the group of sesquiterpene [13,40], is a downstream product of the mevalonate (MVA) and methylerythritol 4-phosphate (MEP) biosynthesis pathway in plants [12,41]. In our dataset, most of the critical enzymes involved in the pathways mentioned above, such as hydroxymethylglutaryl-CoA synthase (HMGS), mevalonate kinase (MK), phosphomevalonate kinase (PMK), 1-deoxy-d-xylulose-5-phosphate synthase (DXS), 1-deoxy-d-xylulose-5-phosphate reductoisomerase (DXR), 4-diphosphocytidyl-2-C-methyl-d-erythritol kinase (CMK), 2-C-methyl-d-erythritol 2,4-cyclodiphosphate synthase (MDS), and 4-hydroxy-3-methylbut-2-enyl diphosphate reductase (HDR) were identified. Bibenzyl compounds are usually synthesized using substrate (L-Phenylalaine) via cinnamic acid with phenylalanine ammonia-lyase (PAL) catalysis (Figure 4). The catalyzation of trans-cinnamate 4-monooxygenase (C4H) resulted in the two isomers of m-coumaric acid and p-coumaric acid. Along with the phenylpropanoid pathway using m-coumaric acid as substrate dihydro-m-Coumaric acid, dihydro-m-Coumaroyl-CoAic and 3,3’5-Trihydrobibenzyl were subsequently synthesized with CYP450, 4-coumarate-CoA ligase (4CL), and bibenzyl synthase (BBS) catalysis, respectively. Our identified DEGs reveal that 11 genes, including two phenylalanine ammonia-lyase (PALs) (LOC110113904/LOC110115785), two trans-cinnamate 4-monooxygenase (C4H) (LOC110113575/LOC110101902), two CYP450s [84A1 and 98A2] (LOC110097166/LOC110101632), two bibenzyl synthases, and one bibenzyl synthase-like (BBS) (LOC110115249, LOC110105072, LOC110105073), two 4CL including one 4-coumarate-CoA ligase 1-like and one 4-coumarate--CoA ligase 2-like (4CL) (LOC110116024/LOC110107453) were significantly up-regulated via MeJA treatment. These genes play critical roles and are closely associated with the pathway of bibenzyl in *D. officinale*. Furthermore, genes potentially involved in flavonoid and phenylpropanoid biosynthesis were also identified. The CYP450 gene family was selected for subsequent analyses due to its critical role in the biosynthesis of secondary metabolites.

### 2.5. Identification, Phylogenetic Analysis, and Classification of CYP450 Gene Family in Dendrobium

**Figure 5:**
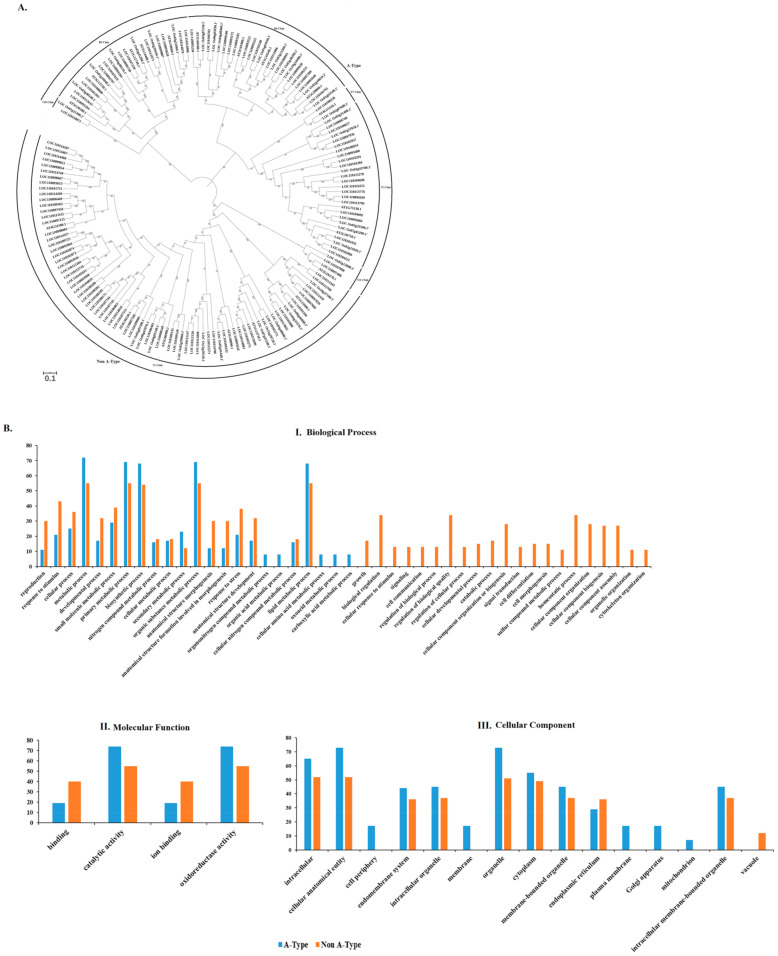
(**A**) Phylogenetic analysis and representative members of the CYP450s genes in *D. officinale*. Tree was constructed using Neighbor-joining with 1000 bootstrap replicates. The code used for different plant CYP450 sequences is AT-*Arabidopsis thaliana*, LOC-*Dendrobium officinale*, ‘LOC OS-*Oryza sativa*. (**B**) Gene ontology annotation of *D. officinale* CYP450 A-type and Non A-type genes. The GO annotation allows genes to be classified into three functional groups, including I. Biological processes, II. Molecular function, and III. Cellular components.

We identified 124 putative CYP450s genes in our *Dendrobium* transcriptome dataset with the three signature motifs of CYP450 genes. Seven CYP450s groups were identified in our phylogenetic dataset, genes belonging to the same group clustered as one clade, and the distribution includes the CYP71 group (68 members belonging to 10 families), the CYP85 group (16 members belonging to 4 families), the CYP72 group (20 members from 5 families), the CYP86 group (16 members from 3 families), the CYP97 group (1 member of 1 family), the CYP710 group (1 member of 1 family), and the CYP711 group (2 members of 1 family). The predicted CYP450s genes from our *Dendrobium* transcriptome results (Figure 5A) were categorized into two main types, A-type (54.8%) and non-A-type (45.2%). They were further classified into 25 families and 25 subfamilies (Supplementary Table S2), respectively.

### 2.6. Functional Annotation of Dendrobium officinale CYP450 Genes

Functional annotation of CYP450 genes was performed based on our root transcriptomic data using Blast2GO [42]. All the predicted 124 genes were assigned to one or more subclasses of GO terms. Biosynthetic processes, organic substance metabolic process, and primary metabolic process were the most common subclasses of biological process. Ion binding and oxidoreductase activity were the most common subclasses in molecular function, while cellular anatomical entity and organelle were the most common in cellular components, respectively (Figure 5B). Specifically, one CYP450 gene (LOC110097166) was involved in various biosynthetic and secondary metabolic processes. Functional annotation of A-type and non-A type P450 genes indicated no significant difference between the two groups (Figure 5B).

### 2.7. KEGG Pathway Analysis of D. officinale CYP450 Genes

*D. officinale* CYP450s were also assigned to 10 KEGG pathways, as indicated in Supplementary Table S3. The KEGG analysis verified that LOC110097166 belongs to a group of Sesquiterpenoid and triterpenoid biosynthesis, responsible for the biosynthesis of bibenzyl.

### 2.8. qRT-PCR Verification

**Figure 6:**
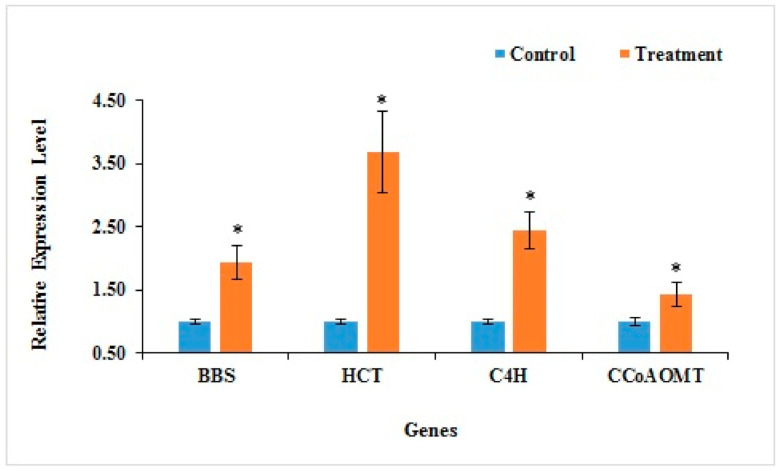
Quantitative real-time PCR analysis of four unigenes associated with the bibenzyl bioScheme 1. Each bar shows the mean ± SE of triplicate assays. The asterisk (*) above the columns indicates a significant change of expression level between the control and treatment at *p* < 0.05 according to the ANOVA tests.

To empirically validate the expression changes generated from our high-throughput RNA-seq, we randomly selected four candidate genes encoding proteins involved in the bibenzyl biosynthesis pathway [LOC110113575 (C4H), LOC110105072 (BBS), LOC110092466 Caffeoyl-CoA O-methyltransferase (CCoAOMT), and LOC110092996 Hydroxycinnamoyl-CoA shikimate/quinate hydroxycinnamoyltransferase (HCT)]. Expressional changes were examined using the qRT-PCR technique. According to the transcriptomic data, the four candidate genes exhibited significant differential expressions between the control and treatment. Results from the qRT-PCR analysis (Figure 6) show that the expression patterns of the four genes were highly consistent with our transcriptome sequencing data and were significantly up-regulated under the MeJA treatment. This result validates that MeJA induced bibenzyl biosynthesis.

## 3. **Discussion**

*Dendrobium* plants are highly prized and have been used as traditional Chinese herbal medicine for many years. The bioactive constituents include polysaccharides, alkaloids, flavonoids, and bibenzyl compounds and are complex to authenticate for drug development [43,44]. Polysaccharides perform immunomodulatory and hepato-protective activities, while bibenzyl compounds exhibit antioxidant, anticancer, and immunomodulatory activities [45,46,47]. Several studies have been conducted to identify putative genes involved in polysaccharide biosynthesis [9,40,48]. However, little is known regarding the physiological and molecular bases of bibenzyl biosynthesis in plants. To our knowledge, this study is the first investigation into the biosynthesis of bibenzyl at the physiological and molecular levels. Bibenzyl compounds are composed of different structural formulas with a pair of benzyl radicals. The erianin and gigantol are structurally similar, and they are represented as bibenzyl compounds [46,47,49,50]. As a result, in this study, the contents of erianin and gigantol were measured as representatives of bibenzyl compounds.

A recent study detected the total alkaloid content of *D. officinale* in the leaf and found a significant increase after exogenous MeJA treatment [51]. In contrast, in our study, we found that bibenzyl compounds (erianin and gigantol) mainly accumulate in the roots and the basal parts of the stem tissues. This suggests that the roots of *Dendrobium* plants may be more important in the extraction of bioactive ingredients of antioxidant and anticancer compounds than other tissues. Many studies have found that the biosynthesis of secondary metabolites (such as terpenoids, phenylpropanoids, flavonoids, and alkaloids) could be induced by JA signaling [51,52,53]. As expected, the accumulation of bibenzyl was induced by the exogenous hormone MeJA in our study. The content of bibenzyl significantly increased between 24 to 36 hours after MeJA treatment, suggesting rapid accumulation of bibenzyl during this timeframe. We reasonably assumed that most genes involved in bibenzyl biosynthesis were actively expressed. Thus, we compared global gene expressions between the root tissues after 24-hour treatment with MeJA and the control (untreated).

Although several *Dendrobium* transcriptomic datasets are available [9,48,51,54], our study annotated 23,131 unigenes, a smaller number than in the studies mentioned above. However, it is comparable to the unigenes identified by Chen et al. [55] in root tissues. In total, 1324 DEGs (912 up-regulated and 430 down-regulated) were identified by comparing expression changes between our two libraries. In our study, fewer genes were identified compared to a recent investigation of transcriptomic analyses using exogenous MeJA treatment in *D. officinale* leaves by Chen et al. [51]. The fewer identified unigenes and DEGs in our study are likely due to differences in tissues tested between these studies (only root tissues were investigated in this study, whereas other studies investigated either leaves, stem, or mixed tissues). We also identified DEGs enriched in the JA signaling pathway and biosynthesis of secondary metabolites. The induced DEGs were significantly enriched in the GO terms related to the diverse processes of secondary metabolites (including sesquiterpene and flavonoid biosynthesis) and in response to JA induction in the signaling pathway. As expected, many DEGs involved in the JA signal pathway were identified, such as PLD, DAD1, LOX, and AOS. In particular, JAZ and MYC are well-known to respond to exogenous MeJA treatment in plants [56,57,58,59,60,61].

The KEGG analysis provides a basis for understanding the functions of the *D. officinale* CYP450 gene in respect to the biosynthesis of secondary metabolites such as bibenzyl. In our *D. officinale* transcriptomic dataset, 124 unigenes were annotated to CYP450. Among them, LOC110097166 [84A1] may be the critical gene responsible for regulating bibenzyl content in *D. officinale*. The involvement of phenylpropanoid biosynthesis in numerous critical biological processes, such as secondary metabolite synthesis, is essential for plant growth [62]. CYP84A1 is a crucial enzyme in the biosynthesis of phenylpropanoid required for the biosynthesis of a wide variety of soluble specific plant metabolites [63]. This branch’s first step (phenylpropanoid) is catalyzed by the CYP450 enzyme ferulate5-hydroxylase (F5H or CYP84A1), which transforms coniferaldehyde and coniferyl alcohol into 5-hydroxylated derivatives [64].

Generally, sesquiterpenes are derived from farnesyl diphospate (FPP) provided by the MVA and MEP pathways in the initial stage of sesquiterpenes biosynthesis in plants [41]. Recently, Chen et al. [51] identified several genes involved in FPP biosynthesis. These identified genes usually function at the initial stages of sesquiterpenes biosynthesis, and most of these identified genes such as HMGS, MK, PMK, DXS, DXR, CMK, MDS, and HDR appeared in our data, signifying that these genes participated in the regulation of the initial biosynthesis of bibenzyl in *D. officinale*. It is possible that the genes (or some of them) identified in our study may be rate-limiting for bibenzyl biosynthesis, and the expression levels of these genes may result in variation of bibenzyl content in various tissues. These identified enzymes could provide valuable genetic resources for future research toward increasing bibenzyl content by modifying their expression levels using genetic engineering techniques. Many differentially expressed TFs (such as bHLH, AP2, and WRKY) were also identified in this study. Previous studies have shown that these TF families, such as the bHLH, AP2, and WRKY families, are involved in various steps of the alkaloid biosynthesis pathways [51,65]. However, whether their different expressions are directly associated with the regulation of bibenzyl biosynthesis remains unknown. Although the biosynthesis pathway of bibenzyl might be conserved in plants, whether the identified putative genes related to bibenzyl biosynthesis are species-specific in *D. officinale* remains uncertain in this study.

## 4. Materials and Methods

### 4.1. Plant Material and Sample Collection

Three-year-old *D. officinale* plants were collected from the Experimental Base of *Dendrobium* Breeding and Planting, located in San jia Cun, Simao town, Puer City, Yunnan Province (latitude 22°47′13″ N; longitude 100°58′37″ E; Altitude: 1342.2 m above sea level). The collection site is a branch of *Dendrobium* domestication unit of the Kunming Institute of Botany, Chinese Academy of Sciences, Kunming, China. The collected samples were formally identified by Professor Li Shu Yun, a taxonomist from Kunming Institute of Botany, Yunnan Province, China. The cultivated samples used in this study were collected in compliance with the institutional guidelines. No voucher specimens were collected and deposited during the sample collection.

### 4.2. Extraction of Plant Materials and Analysis of Bibenzyl Content

*D. officinale* specimens were kept in a greenhouse at the Kunming Institute of Botany Botanical Garden, Chinese Academy of Sciences, in Yunnan Province, China, under day 24 °C and night 18 °C, with natural light. To inspect the changes of bibenzyl compounds in various tissues, we investigated the contents of representative compounds of bibenzyl (erianin and gigantol) among leaf, root, basal stem, and upper stem tissues from three-year-old individuals. Fresh dissected samples were oven-dried at 55 °C until a constant weight was attained and then subsequently pulverized. The pulverized tissues (2 g) of each sample were accurately weighed and refluxed twice with 200 mL of 80% ethanol in a water bath at 80 °C for 2 h. Extracts were concentrated and dried via evaporation; they were re-dissolved in a methanol-to-water ratio of 80:20 (*v*/*v*). The solutions were then subjected to MCI gel to determine the active compounds and eluted with 70% ethanol. The eluted fractions were dried via evaporation and finally dissolved in 25 mL of absolute methanol. Before liquid chromatography–mass spectrometry (LC–MS), 1 mL of the solution was passed through a 0.22 μm microporous membrane. Extracts were analyzed with an Agilent ZORBAX SB-C18 column (4.6 × 50 mm, 2.7 μm) using Liquid Chromatography/Quadrupole Time-Of-Flight Mass Spectrometry (Agilent 1290/6530). This mobile phase consisted of using a methanol-to-water ratio of 80:20 (*v*/*v*) at a flow rate of 500 μL/min. The detection wavelength and column temperature were set at 230 nm and 30 °C. ESI/MS spectra in both positive and negative ion modes were also performed. The isolation width for isolating the precursor ion was 1 to 3 m/z, and the collision energy was 25 to 45%. Standard references of bibenzyl (erianin and gigantol) were prepared with an accurately known concentration of 0.005 mg/mL to identify and quantitate the compounds. Origin Pro software (version 8.5, OriginLab Corporation, Northampton, MA, USA) was used to perform one-way ANOVA and Tukey test at *P* < 0.05 significance level. Error bars representing the standard deviation were derived from each sample in triplicate.

### 4.3. MeJA Treatment Conditions

*D. officinale* plants were divided into two groups: (a) treatment group [MJ] and (b) control group [CT]. The treatment group plants were sprayed with 0.2, 0.5, and 1.5 mM MeJA (dissolved in absolute ethanol and water), while the control group plants were sprayed with only ethanol and water solution (three biological replicates were used per treatment). Tissues including root, basal stem, upper stem, and leaves were harvested at 0 h, 24 h, 36 h, and 48 h of treatment, and immediately frozen in liquid nitrogen before being stored at −80 °C for metabolite and RNA extraction.

### 4.4. RNA Extraction, cDNA Library Preparation, and Transcriptome Sequencing

Total RNA was isolated from the sampled roots of three biological replicates with MeJA (0.5 mM) treatment after 24 hours and another three biological replicates with ethanol and water solution as controls using the RNAprep pure Tissue Kit (Tiangen, Beijing, China), following the manufacturer’s protocol. The RNA quality and purity were verified using 2100 Agilent Bioanalyzer and Qubit 2.0. Equal quantities of total RNA from three biological replicate samples were mixed to prepare the pooled RNA sample for cDNA synthesis to ensure that we obtained full transcriptome. Transcriptome sequencing was performed at the Shanghai Ouyi Biomedical Technology Co., Ltd., Shanghai, China. The pooled mRNA was enriched with Oligo (dT) beads (Thermo Fisher Scientific, Waltham, MA, USA) and fragmented into short sequences from 200 to 400 bp. The cleaved RNA fragments were reverse-transcribed into double-stranded cDNA using random hexamer primers and then purified and ligated to sequencing adapters. The products were purified and enriched by PCR to generate the final cDNA library. Libraries were constructed using TruSeq Stranded mRNA LTSample Prep Kit (Illumina, San Diego, CA, USA) and sequenced on the HiSeqTM 2500 Illumina sequencing platform following the manufacturer’s instructions.

### 4.5. Illumina Sequencing Data Analysis

To improve the sequence quality, reads with poly-N and low-quality fragments were removed to obtain high-quality reads. The clean reads were mapped onto the reference genome of the species (ftp://ftp.ncbi.nlm.nih.gov/genomes/all/GCF/001/605/985/GCF_001605985.1_ASM160598v1/GCF_001605985.1_ASM160598v1_genomic.fna.gz (9 February 2021)) using HISAT2 [66]. The fragments per kilobase per million map reads (FPKM) were calculated by Cufflinks [67] to estimate the level of gene expression.

### 4.6. Analysis of Differentially Expressed Genes (DEGs)

DESeq [68,69] R package was used to determine differential expression of the same genes in the two samples (CT and MJ). In achieving this, two criteria were selected: one was FoldChange, which is the multiple of change in the expression level of the same gene in the two samples; the other was *p*-value < 0.05 or False Discovery Rate (FDR, adjusted *p*-value), which was set as the threshold for significantly differential expression. Biological coefficient of variation (BCV), with a dispersion value of 0.1, was adopted to deal with the absence of biological relicates in this study. The entire design matrix was reduced to a single column for the intercept and dispersion from the reduced model was estimated and inserted into the data object containing the full design matrix. Model fitting and testing were done using glmFit and glmLRT. Unigenes were searched against the flavonoid biosynthetic pathway to identify genes related to bibenzyl biosynthesis.

### 4.7. Functional Annotation

Functional annotations were performed as outlined by Zhang et al. [48]. This was conducted by sequence comparison with public databases, which includes the non-redundant NCBI nucleotide database, the non-redundant protein database, the Swiss-Prot database, and the KOG database using BLASTN and BLASTX with an e-value of 1e-5. The compiled genes selected were grouped into three different groups of biological processes, cellular components, and molecular function using Blast2GO (V2.6.4) (http://www.blast2go.org/(9 February 2021)) [42]. KEGG was used to examine the predicted CYP450 genes and map a potential pathway for a biological understanding of systemic functions (http://www.genome.jp/kegg/ (9 February 2021)) [70].

### 4.8. Alignment and Phylogenetic Study of the Gene Family Dendrobium CYP450

Multiple sequence alignment with the default parameters was done using ClustalX 2.0 software [71]. CYP450 protein sequences from *Arabidopsis thaliana*, *Oryza sativa,* and *D. officinale* were used. CYP450 genes with defined functions within a family were preferentially selected. Phylogenetic tree was developed using the neighbor-joining algorithm [72] with the Poisson model, pair deletion, and bootstrap analysis at 1000 replicates of resampling as per MEGA X [73]. The bootstrap consensus tree was visualized and modified using the https:/itol.embl.de/ (9 February 2021) [74] Interactive Tree of Life (ITOL).

### 4.9. Classification and Characterization of CYP450 Genes in Dendrobium

Based on the amino acid sequence similarity percentage (>40 percent, >55 percent, or >95 percent), identified *Dendrobium* CYP450s were further classified into different families and subfamilies [75].

#### Validation of Genes Related to Bibenzyl Biosynthesis by qRT-PCR

Four genes potentially involved in the bibenzyl biosynthesis pathway were selected for Real-Time PCR experiments. These four genes include bibenzyl synthase-like (BBS), tryptamine hydroxycinnamoyltransferase 1-like (THT1), trans-cinnamate 4-monooxygenase (C4H), and putative caffeoyl-CoA O-methyltransferase (CAMT). Primers were designed using the Primer Premier V5.0 software. All gene IDs and their primer sequences are listed in Supplementary Table S4. Total RNA was isolated from the root samples using the RNAprep pure Tissue Kit (Tiangen, Beijing, China) in compliance with the manufacturer’s protocol. RNA integrity and quality were evaluated with 1.0% formaldehyde agarose gel and Nano-drop 2000 spectrophotometer (Thermo scientific). First-strand cDNA was synthesized using one microgram of RNA in reverse transcription following the manufacturer’s instructions using TransScript All-in-One First-Strand cDNA Synthesis SuperMix for qPCR kit (TransGen Biotech, Beijing, China). All primer pairs were examined using standard real-time PCR. The presence of a single amplification product of the expected size for each gene was verified by electrophoresis on a 1.0% agarose gel with ethidium bromide staining. We performed quantitative real-time (qRT-PCR) using TransStart Tip Green qPCR SuperMix (TransGen Biotech, Beijing, China) on the Bio-Rad CFX96 system (Bio-Rad, USA) as follows: 95 °C for 30 s initial denaturation, followed by 40 cycles of denaturation at 95 °C for 5 s and annealing at 60 °C for 30 s, extension at 72 °C for 30 s. The total volume of the reaction mixture was 20 µL, which contained 2 µL cDNA, 0.4 µL of both Forward and Reverse primer, 10 µL 2 × PerfectStartTM Green qPCR SuperMix, and 7.2 µL of dH20. Amplicons were subjected to melting curve analysis to determine amplification specificity. A melting curve was generated for each sample at the end of each run to assess the amplified products’ purity. The relative levels of expression were calculated using the 2−ΔΔCt method [76] and normalized with actin as the internal control in *D. officinale*. The expression levels of the control samples were normalized to 1. Analyses of qRT-PCR were carried out with three independent biological repetitions to validate our transcriptomic data. Origin Pro software (version 8.5, OriginLab Corporation, Northampton, MA, USA.) was used to perform one-way ANOVA test at *p* < 0.05 significance level.

## 5. **Conclusions**

Based on our investigation on the bibenzyl accumulation in various tissues, we found that bibenzyl compounds mostly accumulate in the root tissues of *D. officinale*. Our transcriptomic analyses resulted in 1342 DEGs, most of which are functionally involved in the JA signaling pathway and secondary metabolites’ biosynthetic processes. In particular, we identified 11 genes in the route of bibenzyl biosynthesis that may play a critical role in regulating bibenzyl biosynthesis in *D. officinale*. This study not only aids our understanding of unique genes involved in the synthesis of bibenzyl in *D. officinale*, but also provides insights into the identification of putative genes associated with bibenzyl biosynthesis and accumulation in *Dendrobium* plants.

## Figures and Tables

**Table 1:** Comparison of bibenzyls contents (erianin and gigantol) in different tissues of *D. officinale* (Mean ± Standard error). ND denotes “not detectable”.

Compounds	Erianin (µg/g)	Gigantol (µg/g)	Total Content (µg/g)
Roots	2.63 ± 0.69	37.01 ± 2.16	39.64 ± 4.14
basal stems	0.61 ± 0.01	22.67 ± 0.15	23.28 ± 0.25
upper stems	ND	12.15 ± 1.87	12.15 ± 1.87
Leaves	ND	ND	ND

## Data Availability

The sequencing data were deposited in the NCBI Sequence Read Archive database as files of SRR9866323 (https://trace.ncbi.nlm.nih.gov/Traces/sra/?run=SRR9866323 (9 February 2021)) and files of SRR9866324 (https://trace.ncbi.nlm.nih.gov/Traces/sra/?run=SRR9866324 (9 February 2021)).

## Supplementary Materials

The following are available online at https://www.mdpi.com/article/10.3390/plants10040633/s1, Supplementary Figure S1: Identification of differentially expressed genes (DEGs). The dots in gray indicate genes that are not differentially expressed, and dots in red denote the up-regulated and down-regulated DEGs, respectively. Supplementary Table S1: GC content showing the percentage of raw reads generated from the CT and MJ libraries with the Q30 percentages as well as the clean and uniquely mapped reads among different treatments. Supplementary Table S2: Characteristics of the predicted CYP450s gene family from the root of *D. officinale*. Supplementary Table S3: *D. officinale* CYP450s Kyoto Encyclopedia of Genes and Genomes (KEGG) pathway distributions. Supplementary Table S4: List of Primer Sequences Used.

## References

[1] Dixon R.A. (2001). Natural products and plant disease resistance. Nature.

[2] Kennedy D.O., Wightman E.L. (2011). Herbal extracts and phytochemicals: Plant secondary metabolites and the enhancement of human brain function. Adv. Nutr..

[3] Kliebenstein D.J., Osbourn A. (2012). Making new molecules—Evolution of pathways for novel metabolites in plants. Curr. Opin. Plant Biol..

[4] Goossens A.H., Laakso S.T., Seppanen-Laakso I., Biondi T., De Sutter S., Lammertyn V., Nuutila F., Soderlund A.M., Zabeau H., Inze M. (2003). A functional genomics approach toward the understanding of secondary metabolism in plant cells. Proc. Natl. Acad. Sci. USA.

[5] Hussain M.S.F., Ansari S., Rahman S., Ahmad M.A., Saeed I.Z. (2012). Current approaches toward production of secondary plant metabolites. J. Pharm. Bioallied Sci..

[6] Yang D.L., Yang J., Mei C.S., Tong X.H., Zeng L.J., Li Q., Xiao L.T., Sun T.P., Li J., Deng X.W. (2012). Plant hormone jasmonate prioritizes defense over growth by interfering with gibberellin signaling cascade. Proc. Natl. Acad. Sci. USA.

[7] Wu Z.Y., Raven P.H., Hong D.Y. (2010). Flora of China. Harv. Pap. Bot..

[8] Yang S. (2016). Comprehensive Utilization of Citrus By-Products. Methods for Determining the Functional Components of Citrus Peel.

[9] Zhang J., He C., Wu K., Teixeira da Silva J.A., Zeng S., Zhang X., Yu Z., Xia H., Duan J. (2016). Transcriptome Analysis of *Dendrobium officinale* and its Application to the Identification of Genes Associated with Polysaccharide Synthesis. Front. Plant Sci..

[10] Tang H., Zhao T., Sheng Y., Zheng T., Fu L., Zhang Y. (2017). *Dendrobium officinale* Kimura et Migo: A Review on Its Ethnopharmacology, Phytochemistry, Pharmacology, and Industrialization. Evid. Based Complementary Altern. Med. eCAM.

[11] Kreis L.M., Carreira E.M. (2012). Total synthesis of (-)-dendrobine. Angew. Chem. Int. Ed. Engl..

[12] Li Q., Ding G., Li B., Guo S.X. (2017). Transcriptome Analysis of Genes Involved in Dendrobine Biosynthesis in Dendrobium nobile Lindl. Infected with Mycorrhizal Fungus MF23 (*Mycena* sp.). Sci. Rep..

[13] Xu J., Zhao W.M., Qian Z.M., Guan J., Li S.P. (2010). Fast determination of five components of coumarin, alkaloids and bibenzyls in *Dendrobium spp*. using pressurized liquid extraction and ultra-performance liquid chromatography. J. Sep. Sci..

[14] Gong Y.Q. (2003). Mechanisms of Erianin Anti-Tumor Angiogenesis.

[15] Barbosa E.G., Bega L.A., Beatriz A., Sarkar T., Hamel E., do Amaral M.S., de Lima D.P. (2009). A diaryl sulfide, sulfoxide, and sulfone bearing structural similarities to combretastatin A-4. Eur. J. Med. Chem..

[16] Li Y., Wang C., Wang F. (2010). Chemical constituents of *Dendrobium candidum*. China J. Chin. Mater. Med..

[17] Su P. (2011). Research on the Molecular Mechanism of Erianin AntiHepatoma Effect.

[18] Cai H.L., Huang X.J., Nie S.P., Xie M.Y., Phillips G.O., Cui S.W. (2015). Study on *Dendrobium officinale* O-acetylglucomannan (Dendronan): Part III-Immunomodulatory activity in vitro. Bioact. Carbohydr. Diet. Fibre.

[19] Zhang X., Xu J.K., Wang J., Wang N.L., Kurihara H., Kitanaka S., Yao X.S. (2007). Bioactive bibenzyl derivatives and fluorenones from *Dendrobium nobile*. J. Nat. Prod..

[20] Hossain M.M. (2011). Therapeutic orchids: Traditional uses and recent advances—An overview. Fitoterapia.

[21] Majumder P.L., Sen S., Majumder S. (2001). Phenanthrene derivatives from the orchid *Coelogyne cristata*. Phytochemistry.

[22] Yahyaa M., Ali S., Davidovich-Rikanati R., Ibdah M., Shachtier A., Eyal Y., Lewinsohn E., Ibdah M. (2017). Characterization of three chalcone synthase-like genes from apple (Malus x domestica Borkh.). Phytochemistry.

[23] Ibdah M., Martens S., Gang D.R. (2018). Biosynthetic Pathway and Metabolic Engineering of Plant Dihydrochalcones. J. Agric. Food Chem..

[24] Jeong Y.J., An C.H., Woo S.G., Park J.H., Lee K.W., Lee S.H., Rim Y., Jeong H.J., Ryu Y.B., Kim C.Y. (2016). Enhanced production of resveratrol derivatives in tobacco plants by improving the metabolic flux of intermediates in the phenylpropanoid pathway. Plant. Mol. Biol..

[25] Peled-Zehavi H., Oliva M., Xie Q., Tzin V., Oren-Shamir M., Aharoni A., Galili G. (2015). Metabolic Engineering of the Phenylpropanoid and Its Primary, Precursor Pathway to Enhance the Flavor of Fruits and the Aroma of Flowers. Bioengineering.

[26] Ferreyra M.L.F., Rius S.P., Casati P. (2012). Flavonoids: Biosynthesis, biological functions, and biotechnological applications. Front. Plant. Sci..

[27] Singh B., Kumar A., Malik A.K. (2017). Flavonoids biosynthesis in plants and its further analysis by capillary electrophoresis. Electrophoresis.

[28] Camila Gomez G.C., Laurent T., Véronique C., Nancy T., Agnès A. (2011). In vivo grapevine anthocyanin transport involves vesicle-mediated trafficking and the contribution of anthoMATE transporters and GST. Plant J..

[29] Jimenez-Garcia S.N.V., Moises A., Guevara-Gonzalez R.G., Torres-Pacheco I., Cruz-Hernandez A., Feregrino-Perez A.A. (2013). Current Approaches for Enhanced Expression of Secondary Metabolites as Bioactive Compounds in Plants for Agronomic and Human Health Purposes—A Review. Polish J. Food Nutr. Sci..

[30] Jia X.L., Wang G.L., Xiong F., Yu X.R., Xu Z.S., Wang F., Xiong A.S. (2015). De novo assembly, transcriptome characterization, lignin accumulation, and anatomic characteristics: Novel insights into lignin biosynthesis during celery leaf development. Sci. Rep..

[31] Zhu J.H., Cao T.J., Dai H.F., Li H.L., Guo D., Mei W.L., Peng S.Q. (2016). De Novo transcriptome characterization of *Dracaena cambodiana* and analysis of genes involved in flavonoid accumulation during formation of dragon’s blood. Sci. Rep..

[32] Kumar A., Kumar S., Bains S., Vaidya V., Singh B., Kaur R., Kaur J., Singh K. (2016). De novo Transcriptome Analysis Revealed Genes Involved in Flavonoid and Vitamin C Biosynthesis in *Phyllanthus emblica* (L.). Front. Plant. Sci..

[33] Pandey S., Goel R., Bhardwaj A., Asif M.H., Sawant S.V., Misra P. (2018). Transcriptome analysis provides insight into prickle development and its link to defense and secondary metabolism in *Solanum viarum* Dunal. Sci. Rep..

[34] Lei Z., Zhou C., Ji X., Wei G., Huang Y., Yu W., Luo Y., Qiu Y. (2018). Transcriptome Analysis Reveals genes involved in flavonoid biosynthesis and accumulation in *Dendrobium catenatum* From Different Locations. Sci. Rep..

[35] Meng Y., Yu D., Xue J., Lu J., Feng S., Shen C., Wang H. (2016). A transcriptome-wide, organ-specific regulatory map of Dendrobium officinale, an important traditional Chinese orchid herb. Sci. Rep..

[36] Yuan Y., Yu M., Jia Z., Song X., Liang Y., Zhang J. (2018). Analysis of Dendrobium huoshanense transcriptome unveils putative genes associated with active ingredients synthesis. BMC Genom..

[37] Mu H.M., Wang R., Li X.D., Jiang Y.M., Wang C.Y., Quan J.P. (2009). Effect of abiotic and biotic elicitors on growth and alkaloid accumulation of *Lycoris chinensis* seedlings. Z. Naturforsch. C.

[38] Wang H., Yu M., Paek K.Y., Piao X.C., Lian M.L. (2016). An efficient strategy for enhancement of bioactive compounds by protocorm-like body culture of *Dendrobium candidum*. Ind. Crop. Prod..

[39] Zhang X.N., Liu J., Liu Y., Wang Y., Abozeid A., Yu Z.G., Tang Z.H. (2018). Metabolomics Analysis Reveals that Ethylene and Methyl Jasmonate Regulate Different Branch Pathways to Promote the Accumulation of Terpenoid Indole Alkaloids in *Catharanthus roseus*. J. Nat. Prod..

[40] Shen C., Guo H., Chen H., Shi Y., Meng Y., Lu J., Feng S., Wang H. (2017). Identification and analysis of genes associated with the synthesis of bioactive constituents in Dendrobium officinale using RNA-Seq. Sci. Rep..

[41] Schwab W., Wust M. (2015). Understanding the Constitutive and Induced Biosynthesis of Mono- and Sesquiterpenes in Grapes (Vitis vinifera): A Key to Unlocking the Biochemical Secrets of Unique Grape Aroma Profiles. J. Agric. Food Chem..

[42] Conesa A., Gotz S. (2008). Blast2GO: A comprehensive suite for functional analysis in plant genomics. Int. J. Plant. Genom..

[43] Ng T.B., Liu J., Wong J.H., Ye X., Wing Sze S.C., Tong Y., Zhang K.Y. (2012). Review of research on Dendrobium, a prized folk medicine. Appl. Microbiol. Biotechnol..

[44] Li Y., Li F., Gong Q., Wu Q., Shi J. (2011). Inhibitory effects of Dendrobium alkaloids on memory impairment induced by lipopolysaccharide in rats. Planta Med..

[45] Ge Y.H., Wang J., Yang F., Dai G.H., Tong Y.L. (2014). Effects of fresh Dendrobium officinale polysaccharides on immune function in mice with Lewis lung cancer. Zhejiang J. Tradit. Chin. Med..

[46] Li M., He Y., Peng C., Xie X., Hu G. (2018). Erianin inhibits human cervical cancer cell through regulation of tumor protein p53 via the extracellular signal-regulated kinase signaling pathway. Oncol. Lett..

[47] Gong Y.Q., Fan Y., Wu D.Z., Yang H., Hu Z.B., Wang Z.T. (2004). In vivo and in vitro evaluation of erianin, a novel anti-angiogenic agent. Eur. J. Cancer.

[48] He C., Zhang J., Liu X., Zeng S., Wu K., Yu Z., Wang X., Teixeira da Silva J.A., Lin Z., Duan J. (2015). Identification of genes involved in biosynthesis of mannan polysaccharides in Dendrobium officinale by RNA-seq analysis. Plant. Mol. Biol..

[49] Charoenrungruang S., Chanvorachote P., Sritularak B., Pongrakhananon V. (2014). Gigantol, a bibenzyl from Dendrobium draconis, inhibits the migratory behavior of non-small cell lung cancer cells. J. Nat. Prod..

[50] Unahabhokha T., Chanvorachote P., Sritularak B., Kitsongsermthon J., Pongrakhananon V. (2016). Gigantol Inhibits Epithelial to Mesenchymal Process in Human Lung Cancer Cells. Evid. Based Complementary Altern. Med. eCAM.

[51] Chen Y., Wang Y., Lyu P., Chen L., Shen C., Sun C. (2019). Comparative transcriptomic analysis reveal the regulation mechanism underlying MeJA-induced accumulation of alkaloids in *Dendrobium officinale*. J. Plant. Res..

[52] Ge Q., Zhang Y., Hua W.P., Wu Y.C., Jin X.X., Song S.H., Wang Z.Z. (2015). Combination of transcriptomic and metabolomic analyses reveals a JAZ repressor in the jasmonate signaling pathway of *Salvia miltiorrhiza*. Sci. Rep..

[53] Cheong J.J., Choi Y.D. (2003). Methyl jasmonate as a vital substance in plants. Trends Genet..

[54] Guo X., Li Y., Li C., Luo H., Wang L., Qian J., Luo X., Xiang L., Song J., Sun C. (2013). Analysis of the *Dendrobium officinale* transcriptome reveals putative alkaloid biosynthetic genes and genetic markers. Gene.

[55] Chen Y., Li F., Tian L., Huang M., Deng R., Li X., Chen W., Wu P., Li M., Jiang H. (2017). The Phenylalanine Ammonia Lyase Gene LjPAL1 Is Involved in Plant Defense Responses to Pathogens and Plays Diverse Roles in Lotus japonicus-Rhizobium Symbioses. Mol. Plant. Microbe Interact..

[56] Yanfang Y., Kaikai Z., Liying Y., Xing L., Ying W., Hongwei L., Qiang L., Duanfen C., Deyou Q. (2018). Identification and characterization of MYC transcription factors in Taxus sp.. Gene.

[57] De Boer K., Tilleman S., Pauwels L., Vanden Bossche R., De Sutter V., Vanderhaeghen R., Hilson P., Hamill J.D., Goossens A. (2011). APETALA2/ETHYLENE RESPONSE FACTOR and basic helix-loop-helix tobacco transcription factors cooperatively mediate jasmonate-elicited nicotine biosynthesis. Plant J..

[58] Toledo-Ortiz G., Huq E., Quail P.H. (2003). The Arabidopsis basic/helix-loop-helix transcription factor family. Plant Cell.

[59] Sun H., Fan H.J., Ling H.Q. (2015). Genome-wide identification and characterization of the bHLH gene family in tomato. BMC Genom..

[60] Guo X.J., Wang J.R. (2017). Global identification, structural analysis and expression characterization of bHLH transcription factors in wheat. BMC Plant Biol..

[61] Niu X., Guan Y., Chen S., Li H. (2017). Genome-wide analysis of basic helix-loop-helix ( bHLH) transcription factors in *Brachypodium distachyon*. BMC Genom..

[62] Du H., Ran F., Dong H.L., Wen J., Li J.N., Liang Z. (2016). Genome-Wide Analysis, Classification, Evolution, and Expression Analysis of the Cytochrome P450 93 Family in Land Plants. PLoS ONE.

[63] Anderson N.A., Bonawitz N.D., Nyffeler K., Chapple C. (2015). Loss of FERULATE 5-HYDROXYLASE Leads to Mediator-Dependent Inhibition of Soluble Phenylpropanoid Biosynthesis in Arabidopsis. Plant Physiol..

[64] Humphreys J.M., Hemm M.R., Chapple C. (1999). New routes for lignin biosynthesis defined by biochemical characterization of recombinant ferulate 5-hydroxylase, a multifunctional cytochrome P450-dependent monooxygenase. Proc. Natl. Acad. Sci. USA.

[65] Yamada Y., Shimada T., Motomura Y., Sato F. (2017). Modulation of benzylisoquinoline alkaloid biosynthesis by heterologous expression of CjWRKY1 in Eschscholzia californica cells. PLoS ONE.

[66] Kim D., Langmead B., Salzberg S.L. (2015). HISAT: A fast spliced aligner with low memory requirements. Nat. Methods.

[67] Roberts A., Trapnell C., Donaghey J., Rinn J.L., Pachter L. (2011). Improving RNA-Seq expression estimates by correcting for fragment bias. Gen. Biol..

[68] Anders S., Huber W. (2012). Differential Expression of RNA-Seq Data at the Gene Level—The DESeq Package.

[69] Anders S., Huber W. (2010). Differential expression analysis for sequence count data. Gen. Biol..

[70] Kanehisa M., Araki M., Goto S., Hattori M., Hirakawa M., Itoh M., Katayama T., Kawashima S., Okuda S., Tokimatsu T. (2008). KEGG for linking genomes to life and the environment. Nucleic Acids Res..

[71] Larkin M.A., Blackshields G., Brown N.P., Chenna R., McGettigan P.A., McWilliam H., Valentin F., Wallace I.M., Wilm A., Lopez R. (2007). Clustal W and Clustal X version 2.0. Bioinformatics.

[72] Saitou N., Nei M. (1987). The neighbor-joining method: A new method for reconstructing phylogenetic trees. Mol. Biol. Evol..

[73] Kumar S., Stecher G., Li M., Knyaz C., Tamura K. (2018). MEGA X: Molecular Evolutionary Genetics Analysis across Computing Platforms. Mol. Biol. Evol..

[74] Letunic I., Bork P. (2016). Interactive tree of life (iTOL) v3: An online tool for the display and annotation of phylogenetic and other trees. Nucleic Acids Res..

[75] Nelson D.R., Koymans L., Kamataki T., Stegeman J.J., Feyereisen R., Waxman D.J., Waterman M.R., Gotoh O., Coon M.J., Estabrook R.W. (1996). P450 superfamily: Update on new sequences, gene mapping, accession numbers and nomenclature. Pharmacogenetics.

[76] Pfaffl M.W. (2001). A new mathematical model for relative quantification in real-time RT-PCR. Nucleic Acids Res..

