# Supplementary material for: Transcriptomic Analyses Shed Light on Critical Genes Associated with Bibenzyl Biosynthesis in Dendrobium officinale
Source: Plants (Basel). 2021 Mar 26;10(4):633. doi: 10.3390/plants10040633 (PMC8065740; doi:10.3390/plants10040633)
Supplement: Supplementary file 1 [file plants-10-00633-s001.pdf]

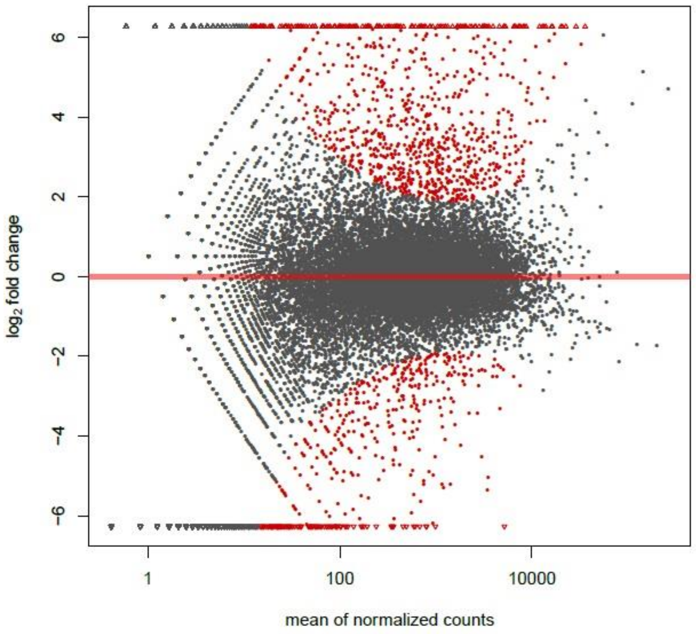

Figure S1. Identification of differentially expressed genes (DEGs). The dots in gray indicates genes that are not differentially expressed and dots in red denote the Up-regulated and down-regulated DEGs, respectively.

Table S1. GC content showing the percentage of raw reads generated from the CK and MJ libraries with the Q30 percentages as well as the clean and uniquely mapped reads among different treatments.

Sample	Raw reads	Clean reads	Uniquely mapped reads	Q30	GC
CK	83,206,690	81,284,898	61,501,743	95.06%	44%
MeJA	82,623,796	81,047,188	47,566,761	95.40%	47%

Table S2. Characteristics of the predicted CYP450s gene family from the root of *D. officinale.*

**Gene**	**Type**	**Clan**	**Family**	**Subfamily**
LOC110092036	A	71	71	CYP71A1
LOC110093613	A	71	71	CYP71A1
LOC110093703	A	71	71	CYP71A9
LOC110093968	A	71	71	CYP71A1
LOC110095064	A	71	71	CYP71A1

LOC110095634	A	71	706	CYP706A2
LOC110096469	A	71	71	CYP71A1
LOC110097125	A	71	71	CYP71A1
LOC110097166	A	71	84	CYP84A1
LOC110097434	A	71	71	CYP71A1
LOC110097959	A	71	89	CYP89A2
LOC110097960	A	71	89	CYP89A2
LOC110098083	A	71	71	CYP71A1
LOC110098549	A	71	93	CYP93D1
LOC110098667	A	71	71	CYP71A1
LOC110099851	A	71	71	CYP71A9
LOC110099854	A	71	71	CYP71A9
LOC110100088	A	71	71	CYP71A1
LOC110100282	A	71	71	CYP71A1
LOC110100290	A	71	71	CYP71A1
LOC110101138	A	71	71	CYP71A1
LOC110101184	A	71	77	CYP77A3
LOC110101632	A	71	98	CYP98A2
LOC110101738	A	71	89	CYP89A2
LOC110101751	A	71	71	CYP71A1
LOC110102873	A	71	71	CYP71A1
LOC110102874	A	71	71	CYP71A1
LOC110103275	A	71	78	CYP78A5
LOC110104859	A	71	71	CYP71A1
LOC110105033	A	71	71	CYP71A1
LOC110105520	A	71	71	CYP71A1
LOC110105775	A	71	78	CYP78A4
LOC110105808	A	71	77	CYP77A1
LOC110106461	A	71	93	CYP93D2
LOC110106863	A	71	71	CYP71D312
LOC110107744	A	71	71	CYP71D7
LOC110107745	A	71	71	CYP71D7
LOC110108171	A	71	71	CYP71D7
LOC110109066	A	71	84	CYP84A1
LOC110109120	A	71	71	CYP71A1
LOC110109133	A	71	71	CYP71A1
LOC110109162	A	71	71	CYP71A1
LOC110109725	A	71	71	CYP71A1
LOC110112139	A	71	81	CYP81E8
LOC110112147	A	71	81	CYP81E8
LOC110112204	A	71	71	CYP71A1
LOC110112271	A	71	81	CYP81D1
LOC110112418	A	71	89	CYP89A2
LOC110112710	A	71	71	CYP71A1
LOC110113345	A	71	89	CYP89A2

LOC110113699	A	71	78	CYP78A5
LOC110113842	A	71	77	CYP77A4
LOC110113995	A	71	71	CYP71D11
LOC110114010	A	71	71	CYP71D8
LOC110114083	A	71	78	CYP78A5
LOC110114269	A	71	71	CYP71A1
LOC110114357	A	71	71	CYP71A1
LOC110114467	A	71	71	CYP71A9
LOC110114468	A	71	71	CYP71A9
LOC110114507	A	71	71	CYP71A9
LOC110114706	A	71	703	CYP703A2
LOC110114744	A	71	71	CYP71A9
LOC110115096	A	71	81	CYP81E8
LOC110105595	A	71	89	CYP89A2
LOC110107553	A	71	71	CYP71D7
LOC110112115	A	71	71	CYP71A6
LOC110113709	A	71	89	CYP89A2
LOC110108199	A	71	71	CYP71D7
LOC110093666	non-A	72	721	CYP721A1
LOC110094269	non-A	72	72	CYP72A15
LOC110095600	non-A	72	749	CYP749A22
LOC110097936	non-A	72	714	CYP714C2
LOC110098748	non-A	72	714	CYP714B3
LOC110101935	non-A	72	734	CYP734A1
LOC110102066	non-A	72	734	CYP734A6
LOC110102957	non-A	72	714	CYP714C2
LOC110103284	non-A	72	749	CYP749A22
LOC110103291	non-A	72	749	CYP749A22
LOC110104221	non-A	72	734	CYP734A6
LOC110104692	non-A	72	721	CYP721A1
LOC110104696	non-A	72	72	CYP72A15
LOC110105571	non-A	72	714	CYP714B3
LOC110108657	non-A	72	714	CYP714B3
LOC110113776	non-A	72	72	CYP72A219
LOC110113799	non-A	72	72	CYP72A219
LOC110115270	non-A	72	72	CYP72A219
LOC110116255	non-A	72	72	CYP72A219
LOC110106854	non-A	72	734	CYP734A1
LOC110092087	non-A	85	724	CYP724B1
LOC110092799	non-A	85	90	CYP90B1
LOC110094184	non-A	85	87	CYP87A3
LOC110094797	non-A	85	90	CYP90D2
LOC110095594	non-A	85	85	CYP85A1
LOC110101203	non-A	85	90	CYP90A1
LOC110107220	non-A	85	87	CYP87A3

LOC110107619	non-A	85	90	CYP90D2
LOC110108808	non-A	85	90	CYP90A1
LOC110112633	non-A	85	85	CYP85A1
LOC110112973	non-A	85	724	CYP724B1
LOC110114079	non-A	85	90	CYP90B2
LOC110114986	non-A	85	90	CYP90B2
LOC110099807	non-A	85	90	CYP90B1
LOC110093620	non-A	85	90	CYP90B2
LOC110096728	non-A	85	87	CYP87A3
LOC110093174	non-A	86	704	CYP704C1
LOC110093175	non-A	86	704	CYP704C1
LOC110093521	non-A	86	94	CYP94B3
LOC110093522	non-A	86	94	CYP94B1
LOC110095250	non-A	86	704	CYP704B1
LOC110095646	non-A	86	86	CYP86A8
LOC110097389	non-A	86	86	CYP86A22
LOC110099100	non-A	86	704	CYP704C1
LOC110099638	non-A	86	86	CYP86B1
LOC110104795	non-A	86	86	CYP86A1
LOC110106702	non-A	86	704	CYP704C1
LOC110108993	non-A	86	86	CYP86B1
LOC110109253	non-A	86	86	CYP86B1
LOC110112006	non-A	86	94	CYP94B3
LOC110116548	non-A	86	94	CYP94C1
LOC110093185	non-A	86	704	CYP704C1
LOC110108258	non-A	97	97	CYP97B2
LOC110110071	non-A	710	710	CYP710A11
LOC110097408	non-A	711	711	CYP711A1
LOC110107898	non-A	711	711	CYP711A1

Table S3. *D. officinale* CYP450s Kyoto Encyclopedia of Genes and Genomes (KEGG) pathway distributions.

**Pathways**	**#Seqs**	**#Enzs**
Sesquiterpenoid and triterpenoid biosynthesis	44	2
Diterpenoid biosynthesis	7	2
Monoterpenoid biosynthesis	6	4
Isoquinoline alkaloid biosynthesis	6	3
Flavonoid biosynthesis	5	2
Phenylpropanoid biosynthesis	3	1
Stilbenoid, diarylheptanoid and gingerol biosynthesis	3	1

Ubiquinone and other terpenoid-quinone biosynthesis	3	1
Flavone and flavonol biosynthesis	2	1
Limonene and pinene degradation	1	1

Table S4. List of Primer Sequences Used.

**Gene Name**	**Primer Sequence**
LOC110105072-F	5' GTTCACCCCTTTTGGCATTAC 3'
LOC110105072-R	5' CTGGCTTCAACCCCATTCTC 3'
LOC110092466-F	5' CTTGCTACAGCTCTGGCCTTAC 3'
LOC110092466-R	5' GTGCTCAACTCCTGCTTCCTT 3'
LOC110113575-F	5' GTGTTGGTTGAGAAGGCGTTAC 3'
LOC110113575-R	5' GGAGCCAGTTGCCGAAGAT 3'
LOC110092996-F	5' CAGATGGTTCGCTTCGGTAA 3'
LOC110092996-R	5' GCTTCGATGGAGGAATCAGTG 3'
ACTIN-F	5'-TCCCAAGGCAAACAGAGAAA-'3
ACTIN-R	5'-GGCCACTAGCATATAGGGAAAG-'3
